# Development of L-Asparaginase Biobetters: Current Research Status and Review of the Desirable Quality Profiles

**DOI:** 10.3389/fbioe.2018.00212

**Published:** 2019-01-10

**Authors:** Larissa Pereira Brumano, Francisco Vitor Santos da Silva, Tales Alexandre Costa-Silva, Alexsandra Conceição Apolinário, João Henrique Picado Madalena Santos, Eduardo Krebs Kleingesinds, Gisele Monteiro, Carlota de Oliveira Rangel-Yagui, Brahim Benyahia, Adalberto Pessoa Junior

**Affiliations:** ^1^Department of Biochemical and Pharmaceutical Technology, School of Pharmaceutical Sciences, University of São Paulo, São Paulo, Brazil; ^2^Department of Chemistry, CICECO, Aveiro Institute of Materials, University of Aveiro, Aveiro, Portugal; ^3^Department of Chemical Engineering, Loughborough University, Loughborough, United Kingdom

**Keywords:** L-asparaginase, quality-by-design, biobetters, protein engineering, PEGylation, nanobiotechnology, acute lymphoblastic leukemia, site-directed mutagenesis

## Abstract

L-Asparaginase (ASNase) is a vital component of the first line treatment of acute lymphoblastic leukemia (ALL), an aggressive type of blood cancer expected to afflict over 53,000 people worldwide by 2020. More recently, ASNase has also been shown to have potential for preventing metastasis from solid tumors. The ASNase treatment is, however, characterized by a plethora of potential side effects, ranging from immune reactions to severe toxicity. Consequently, in accordance with Quality-by-Design (QbD) principles, ingenious new products tailored to minimize adverse reactions while increasing patient survival have been devised. In the following pages, the reader is invited for a brief discussion on the most recent developments in this field. Firstly, the review presents an outline of the recent improvements on the manufacturing and formulation processes, which can severely influence important aspects of the product quality profile, such as contamination, aggregation and enzymatic activity. Following, the most recent advances in protein engineering applied to the development of biobetter ASNases (i.e., with reduced glutaminase activity, proteolysis resistant and less immunogenic) using techniques such as site-directed mutagenesis, molecular dynamics, PEGylation, PASylation and bioconjugation are discussed. Afterwards, the attention is shifted toward nanomedicine including technologies such as encapsulation and immobilization, which aim at improving ASNase pharmacokinetics. Besides discussing the results of the most innovative and representative academic research, the review provides an overview of the products already available on the market or in the latest stages of development. With this, the review is intended to provide a solid background for the current product development and underpin the discussions on the target quality profile of future ASNase-based pharmaceuticals.

## Introduction

L-asparaginase as a chemotherapeutic agent represented a milestone in the field of medicine due to the ratio of acute lymphoblastic leukemia children patients who achieve complete remission after treatment incorporating ASNase (93%) and due to its selectivity against the tumor cells. Its main mechanism of action is the depletion of the amino acid L-asparagine (L-Asn) from the bloodstream, which is hydrolyzed into aspartic acid (ASP) and ammonia (NH_3_). Since they lack the enzyme asparagine synthetase (EC 6.3.5.4), tumor cells are unable to synthesize enough L-asparagine for their maintenance and accelerated growth, which compromises its cellular functions and leads to cell death.

The medical use of ASNase is, however, not without risks, being associated with allergic reactions and several types of toxicity (Mitchell et al., 1994; Nowak-Göttl et al., 1994; Rizzari et al., 2000), hence there is a current need for novel biobetter ASNases in the market. The term biobetter, also called biosuperior, refers to new drugs designed from existing peptide or protein-based biopharmaceuticals by improving their properties such as affinity, selectivity and stability against degradation (Courtois et al., 2015; Lagassé et al., 2017).

The development of biopharmaceutical products with an improved quality profile is one of the guiding principles of the “Quality-by-Design” paradigm. This translates as starting drug development with an application in mind, which means not only, defining the clinical condition the new product is supposed to treat, but also its pharmacokinetics (administration, distribution, metabolism and excretion), pharmacodynamics (mechanism of action) and safety (potential toxicity) (Eon-Duval et al., 2012; Colombo et al., 2018). This is an important paradigm shift from the traditional approach of discovering new molecules first and later finding potential applications for them (Rathore and Winkle, 2009).

The development of production technologies focused on an economically viable and safe ASNase has both social and economic importance. In this context, the implementation of the Quality-by-Design (QbD) philosophy in the product design and process development of this important chemotherapeutic drug is the main long-term goal of the present review. However, in the vast majority of the ASNase production studies available in the current scientific literature, the focus of process optimization was yield and recovery maximization without extensive consideration on quality aspects of the final product. These results are fundamental for the economic viability of ASNase production, but still require more effort on process development aiming at clinical efficacy and safety. In the following pages, the current status of ASNase production technology will be reviewed as well as the most recent advances on product design.

## L-Asparaginase

L-asparaginase (EC 3.5.1.1) (ASNase) is an amidohydrolase, which can hydrolyze both asparagine (L-asparaginase activity) and glutamine (L-glutaminase side activity) (Chan et al., 2014). The mechanism of antitumor action is also associated with the interference on the signaling pathways and inhibition of expression of oncogenic transcription factors (Avramis and Tiwari, 2006). ASNase is used as a biopharmaceutical and is considered one of the most important oncologic drugs, being a key component of the acute lymphoblastic leukemia (ALL) and lymphosarcoma treatment (Margolin et al., 2011).

ALL is the most common type of childhood cancer, however about 4 out of every 10 cases occur in adults (Nguyen et al., 2018) and, according to the estimative made by Solomon et al. (2017), in 2020 approximately 53,000 cases are expected worldwide. Recent studies have also reported the ASNase contribution to the reduction of cancer metastasis. Further developments might be expected as a result of the application of ASNase to reduce cancer invasion, circulation of tumor cells and metastasis as recently reported for a mouse model of breast cancer by Knott et al. (2018). These researchers demonstrated that the amino acid asparagine governs metastasis partly through regulation of a significant cellular process in the metastatic cascade named epithelial-to-mesenchymal transition (EMT), which leads to the expression of mesenchymal properties facilitating metastasis. Moreover, another study revealed that when extracellular glutamine levels drop, tumor cells become dependent on asparagine for proliferation and protein synthesis (Pavlova et al., 2018). Hence, asparagine and glutamine are already considered “co-conspirators” for metastasis, as stated by Luo et al. (2018); in this way, new insights can be scientifically and rationally employed for this new application of ASNase as a biopharmaceutical.

Currently, three ASNase preparations are available; the native asparaginase derived from *Escherichia coli*, a PEGylated form of this enzyme (PEG-asparaginase) and a product isolated from *Dickeya chrysanthemi* (*Erwinia chrysanthemi*) (Merck, 2000; European Medicines Agency, 2015, 2016; Medicines Evaluation Board, 2015). *E. coli* produces two types of ASNase (EcA I and EcA II), that present distinct characteristics. EcA I is a constitutive enzyme found in the cytoplasm whereas EcA II is located in the periplasm of the bacteria and has a higher affinity to L-asparagine (Eca I K_M_ = 3.5 mM and Eca II K_M_ = 10–15 μM) (Yun et al., 2007). Only the EcA II is used for clinical application. ASNase from *Dickeya* (ErA) and from *E. coli* (EcA II) have the same mechanism of action against tumor cells, however their pharmacokinetics, affinity for the substrate (K_M_) and immune system sensitization profile are different. Therefore, the change to ErA is an important option for patients that present allergic response to the treatment with *E. coli* ASNase (EcA II) (the first choice in the ALL treatment protocol) (El-Naggar et al., 2015).

The therapeutic use of ASNase still faces some challenges as several types of allergic reactions occur due to its high immunogenicity as well as clinically important toxicities, such as pancreatitis, thrombotic events, mucositis, nausea, diarrhea, vomiting, liver dysfunction, hyperglycemia, dyslipidemia, neutropenia, coagulopathy, headache, abdominal pain and central nervous system dysfunctions (Mitchell et al., 1994; Nowak-Göttl et al., 1994; Rizzari et al., 2000). Furthermore, ASNase presents low stability in serum and fast plasma clearance due to the action of human proteases and antibodies (Pieters et al., 2012). The search for alternative bioprocesses, enzyme engineering, PEGylation and alternative formulations have been performed in order to solve these problems, as discussed later.

## Biobetters and Biosimilars

**Figure 1:**
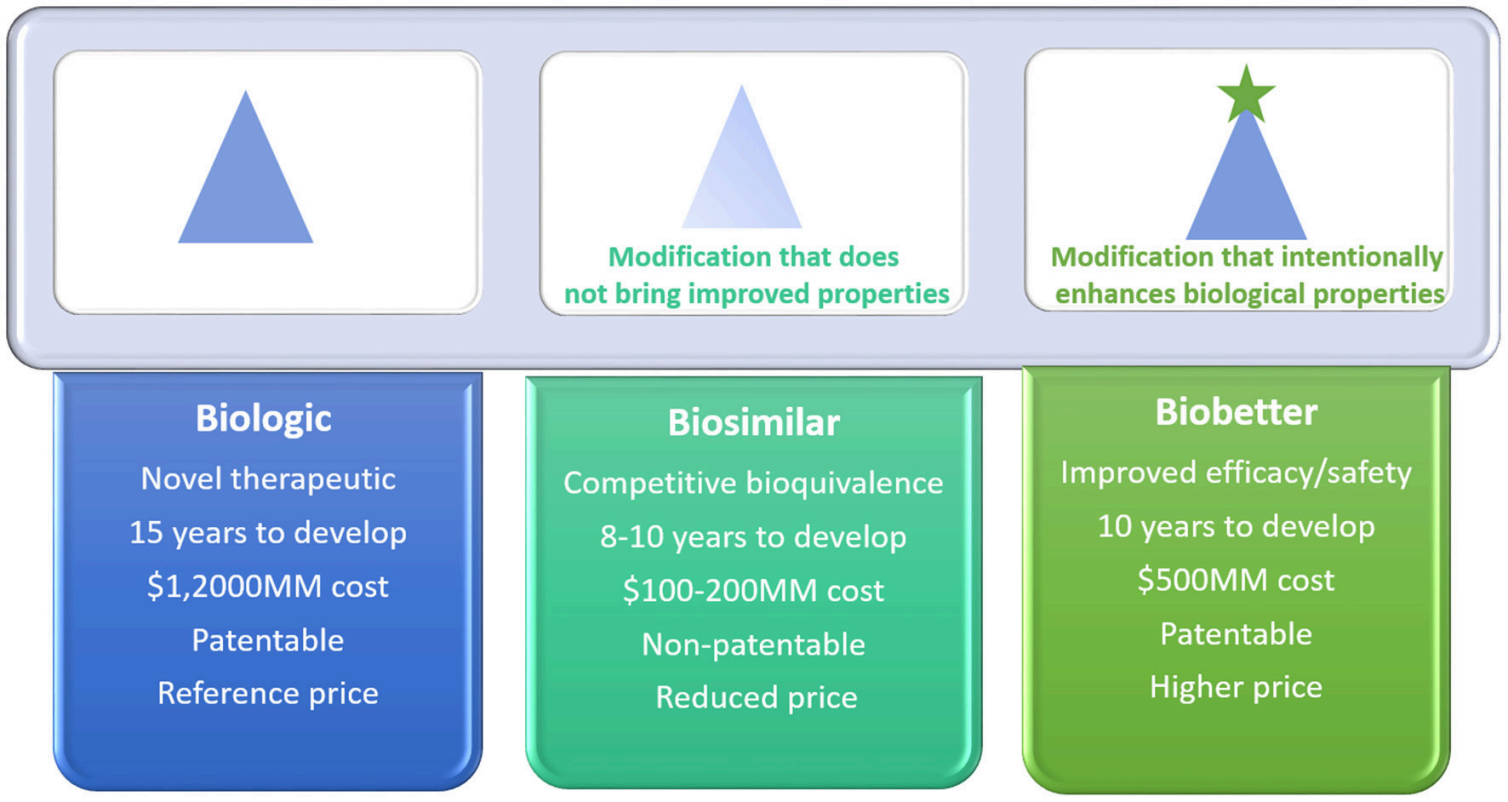
Comparison between biological reference drug, biosimilar and biobetter in terms of development time, overall cost of production, patent protection, and commercial value.

Biobetters are manufactured through chemical or molecular modifications of the originator product by functional changes that may include increased half-life, reduced toxicity, reduced immunogenicity, and enhanced pharmacokinetics and/or pharmacodynamics. This novel category of better biologics emerged over the last few years and gained industrial attention, mainly due to their reduced commercial risk, since they are patentable and worth higher prices due to their clinical advantages (Courtois et al., 2015; Lagassé et al., 2017). Biosimilars, on the other hand, are biologicals with equal efficacy as the originator drug at a reduced price. They are, however, not entitled to patent protection or data exclusivity (Kadam et al., 2016; Sandeep et al., 2016). Biosimilars aim to establish similarity to a known biological. Figure 1 depicts a general comparison between biosimilars and biobetters in terms of their properties and economical/regulatory aspects. In this review the main strategies for the development of ASNase biobetters will be explored by providing an updated overview and highlighting the inherent challenges and opportunities.

## Quality-by-Design

In order to modernize the chemical manufacturing control review process, the U.S. Federal Drug and Food Administration (FDA) published in 2002 the “*Pharmaceutical current good manufacturing practices (CGMPs) for the 21st Century: A Risk-Based Approach*” initiative (FDA, 2004b). The purpose of this initiative was to motivate the pharmaceutical industry to continuously innovate the manufacturing process of drug products and to facilitate the development of new treatments (FDA, 2004a). The FDA initiative was followed by the adoption of similar recommendations by the European Medicines Agency (2018) and by the Japanese Pharmaceutical and Medical Devices Agency (PMDA, 2013) in conformance with the International Conference on Harmonization of Technical Requirements for Registration of Pharmaceuticals for Human Use (ICH, 2005, 2008, 2009, 2012).

While the continuous technological progress could allow immense gains in both quality and productivity for the pharmaceutical industry, the regulatory agencies need to keep close track of production processes in order to safeguard the public against potentially threatening practices. With the new recommendations, the FDA sought to resolve the perceived conflict between continuous process improvement and quality assurance by encouraging manufacturers to develop a deeper and science-based understanding of the relation between process parameters and the characteristics of the final product (Yu, 2008).

Within the new framework, the manufacturers would be granted more freedom to improve manufacturing processes as long as solid knowledge of process variables and their effects on the clinical activity of the final product could be demonstrated. The data collected during process development as well as mechanistic models relating variables and outcomes would then be filed and analyzed by the regulatory agency in order to justify eventual changes in the production process (Rathore, 2009).

Among the recommendations of the FDA initiative, was the adoption of the “Quality-by-Design” (QbD) approach in the development of new drug products. The QbD paradigm was popularized in the early 1990', particularly in the automobile industry, by J. M. Juran and advocates a bottom-up approach for product design, where the customers' needs form the basis of process development (Juran, 1992).

Although facing some initial resistance, the adoption of QbD seems to be gaining track as companies are increasingly adopting its elements in their drug development pipelines, with the oral antihyperglycemic Januvia® (Sitagliptin, Merck & Co, United States) being the first to be approved within the new framework (Woodley, 2018). More recently, Gazyva® (Obinutuzumab, Roche AG, Switzerland) became the first biopharmaceutical developed in conformance with QbD principles to be approved by the FDA for treating chronic lymphocytic leukemia and follicular lymphoma in 2013 (Luciani et al., 2015; Sommeregger et al., 2017).

**Figure 2:**
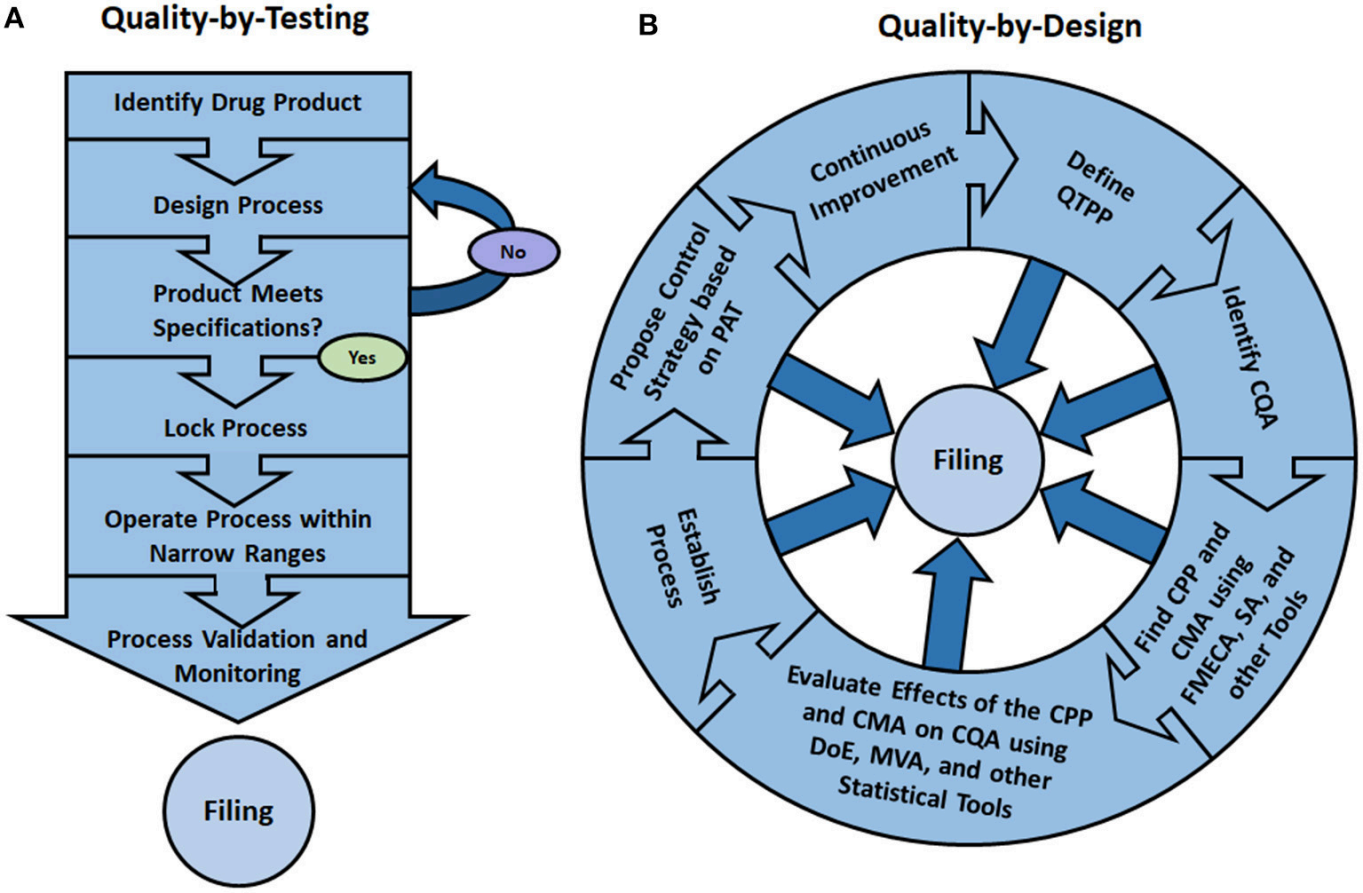
In the traditional “Quality-by-Testing” (QbT) paradigm **(A)**, the prospective drug product is first identified and a manufacturing process is proposed and adjusted until the finished product meets quality specifications. Afterwards, the operating parameters are locked, validated and filed with the regulatory agency. The process is then operated within narrow ranges around the set points, which (ideally) guarantees product consistency. In the “Quality-by-Design” (QbD) paradigm **(B)**, the first step is the definition of the “Quality Target Product Profile” (QTPP) of the prospective pharmaceutical. Afterwards, using risk assessment tools, the “Critical Quality Attributes” (CQA) of the product are identified and, based on them, “Critical Process Parameters” (CPP) and “Critical Material Attributes” (CMA) are found using “Failure Mode Effects and Criticality Analysis” (FMECA), “Sensitivity Analysis” (SA), among other tools. Then, using such statistical tools as “Design of Experiments” (DoE) and “Multivariate Analysis” (MVA), the impact of the CMA and CPP on the CQA are studied, thus allowing process redesign and the removal of quality bottlenecks. Using “Process Analytical Technology” (PAT) a control strategy can then be proposed. Since, within the QbD paradigm, the whole research process is filed with the regulatory agency, the manufacturing process can more easily be improved upon (Rathore, 2014).

As represented in Figure 2A, in the traditional “Quality-by-Testing” paradigm, after the prospective drug product is identified, the process is iteratively redesigned until the finished product meets specifications. After validation, the manufacturer locks the control parameters, files process data and operates within narrow ranges around the set points to guarantee consistency of the final product. Within the QbD paradigm, on the other hand, as shown in Figure 2B, all data and process models developed during the initial research phases are filed with the regulatory agency thus facilitating the approval of continuous process improvements (Rathore, 2014).

In the QbD framework, the desired product characteristics are globally known as its “Quality Target Product Profile” (or QTPP, Figure 2B) (ICH, 2009), which are typically defined in conjunction with the biological activity. This is in contrast with the product “Critical Quality Attributes” (or CQAs), which are surrogate metrics of the product quality profile that serve as basis for quantitative process evaluation (Rathore, 2016). While QTPPs can be described in somewhat loose terms, the CQAs are by definition quantifiable and normally need to fall between certain limits to guarantee product conformance, safety and efficacy (Rathore, 2014). In the spirit of science-based understanding the drug manufacturing process, CQAs are selected from a large spectrum of product quality attributes based on how critical they are for the desired application. It is important, however, that just a few attributes are selected, based on the likelihood and severity of them failing to meet specifications, so that the whole production process development can be geared toward ensuring the safety and effectiveness of the final product (Yu, 2008).

Given that the understanding of the relation between quality attributes and the desired biological activity (i.e., the QTPP) forms the basis of proper CQA selection, a broad experimental practice with the drug product is usually required for QbD-based process development. This need can, however, be minimized when a rich literature describing the product is already available. This is the case for generic and biosimilar products, which follow in the footsteps of extensive research on the innovator drug and whose development can be based on wider population studies (Yu, 2008). The QbD paradigm is, therefore, ideal for the development of the manufacturing process of biosimilar drugs (Kenett and Kenett, 2008; Vulto and Jaquez, 2017). The same principle can, moreover, also be applied for prospecting potential CQAs in the development of biobetter drugs (Jozala et al., 2016) and nanopharmaceuticals (Colombo et al., 2018), which is one of the main focuses of this review.

Aiming at the understanding of product variability and its sources, process development within the QbD paradigm is normally associated with statistical models, such as multivariate analysis (MVA), Design of Experiments (DoE), Monte Carlo simulations, among others, that relate process parameters and material attributes to the CQA (Kenett and Kenett, 2008; Mandenius and Brundin, 2008; Mandenius et al., 2009). A Failure Mode Effects and Criticality Analysis (FMECA) (ICH, 2005) followed by a sensitivity analysis (SA) will often provide a reliable list of the critical material attributes (CMAs) and process parameters (CPPs), which can then be targeted by a tight and robust control strategy (Benyahia et al., 2012; Mascia et al., 2013; Lakerveld et al., 2015). Based on this information, the process can be designed so that the insertion of potential quality bottlenecks in the manufacturing process can be avoided by selecting unit operations that can more reliably deliver the QTPP (Benyahia et al., 2012; Benyahia, 2018). The benefits of such an integrated end-to-end approach were clearly demonstrated through the continuous production of pharmaceuticals (Mascia et al., 2013).

It is not, however, always possible to employ entirely reliable steps in the production process. For this reason, the FDA's “*21st century CGMP initiative”* strongly advocates employing so-called Process Analytical Technology (or PAT) to monitor the variables of the manufacturing process in real time. These methods serve a 2-fold purpose: (1) to develop a deeper understanding of the correlation between process variables and CQAs, and, (2) to allow real time release of a product batch. In alignment with QbD principles, the FDA believes that reduction of overall processing time adds to product quality assurance, which can be achieved by real time decision-making, i.e., real time batch release, based on process data (FDA, 2004a). Furthermore, the implementation of PAT can be the basis for advanced process control, another of the QbD goals.

A variety of ASNase improvements have been recently studied, aiming at reducing its affinity for glutamine, aggregation and immunogenicity and decreasing the concentration of contaminants in the final product as well as increasing its activity, proteolysis resistance and blood serum half-life (Lopes et al., 2017). As it will be shown next, these desired characteristics form the basis of potential innovative products that may soon be available for the patients in need.

## L-Asparaginase Manufacturing: Production Process and Purification

A robust bioprocess is central for guaranteeing that the target quality profile of the final product meets specifications, which represents one of the biggest obstacles for the development of biopharmaceuticals, since small alterations in the process operating parameters, cell line, production methods and purification steps can affect critical quality attributes (CQAs) such as structure, activity and contaminant concentration (Sassi et al., 2015).

ASNase production is divided into upstream and downstream processing. Upstream processing is the transformation of substrates into the product. The upstream process development includes the selection of the cell line, culture media, bioreactor parameters (i.e., pH, temperature, oxygen supply, etc.), process selection (submerged/solid state cultivation, batch, fed-batch, continuous, etc.) and optimization. Downstream processing includes all steps required for the enzyme purification, such as initial recovery, purification and polishing. The downstream process must achieve the removal of host cell protein (HCP), process and products related impurities, DNA, buffer, antifoam agents, aggregates, fragments, among others (Jozala et al., 2016; Lopes et al., 2017). Regarding injectable biopharmaceuticals, such as ASNase, the manufacturing process must be even more judicious, since the presence of contaminants, may potentially lead to detrimental clinical consequences (Zenatti et al., 2018).

### Upstream

#### L-Asparaginase Producing Microorganisms

The selection of the cell line forms the basis of bioprocess development (medium culture, type of process and its parameters, purification strategy, etc.) and affects the characteristic of the enzyme produced, directly influencing the product quality profile. Plants, animals and microorganisms, including bacteria, filamentous fungi and yeast, can produce ASNase. Among them, the microbial enzyme is the most convenient, due to its consistent profile, stability, relative ease production and purification, which simplifies modification and optimization of the manufacturing process, when compared to the plant and animal alternatives (Lopes et al., 2017).

Several ASNase producing microorganisms have already been described in the literature, such as Escherichia coli, Dickeya chrysanthemi (previously known as Erwinia chrysanthemi), Saccharomyces cerevisiae, Aspergillus sp., Serratia marcescens, Proteus vulgaris among others, and screening work continues to find new ones (Rowley and Wriston, 1967; Tosa et al., 1971; Costa et al., 2016; Doriya and Kumar, 2016; Vimal and Kumar, 2017; Qeshmi et al., 2018; Vala et al., 2018). However, as previously mentioned, only the enzymes from E. coli and D. chrysanthemi are produced on an industrial scale for pharmaceutical use (Merck, 2000; European Medicines Agency, 2015, 2016; Medicines Evaluation Board, 2015).

Regarding bacteria, members of the *Enterobacteriaceae* family are the best producers of ASNase. Eukaryotic microorganisms have also been studied for producing enzymes presenting potentially better compatibility with the human immune system due to their evolutionarily proximity (Doriya and Kumar, 2016).

Scientific literature reports many studies of ASNases with improved pharmacokinetics and reduced side effects. More specifically, studies refers to the search for reduced glutaminase activity, lower K_M_ values, lower molecular weights, greater structural diversity and different responses to effector molecules (Krishnapura et al., 2016). Additionally, extracellularly secreted ASNases could greatly simplify downstream processing (Gholamian et al., 2013; Vimal and Kumar, 2017). The first reports on this field describe the use of the Nessler assay, a time consuming and laborious method to detect ASNase activity in culture filtrates based on the release of ammonium by ASNase enzymatic cleavage, which interacts with the Nessler's reagent giving rise to a brown colored compound (Nakahama et al., 1973). More recently, plate assay became widely used since it is fast, sensitive, efficient and reproducible for screening large numbers of microorganisms (Gulati et al., 1997; Mahajan et al., 2013; Dhale and Mohan-kumari, 2014; Meghavarnam and Janakiraman, 2015; Doriya and Kumar, 2016; Vaishali and Bhupendra, 2017). Plate assay screenings are often based on indicators that change color due to the pH increase that results from the ammonium released from ASN hydrolysis (Gulati et al., 1997; Doriya and Kumar, 2016). However, this method has certain limitations such low sensitivity and the fact that it do not measure intracellular ASNase activity (Vaishali and Bhupendra, 2017). After the initial screening, other methods are required for the quantitative evaluation of enzymatic activity such as the aforementioned Nessler's reaction (Peterson and Ciegler, 1969); the L-aspartyl-β-hydroxamic acid (AHA) method, wich evaluates aspartyl β-hydroxamate formation after L-asparagine hydrolysis in the presence of hydroxylamine (Frohweinm et al., 1971); circular dichroism spectroscopy (Kudryashova and Sukhoverkov, 2016); amplex Red method (Karamitros et al., 2014), among others. The screening methods for finding enzymes with reduced glutaminase activity are similar to the plate methods used for ASNase activity detection, i.e., they are based on pH switch, but use GLN instead of ASN as a substrate. However, the determination of K_M_, K_cat_, molecular weight and molecular structure are only possible after purification and require more complex techniques (Mahajan et al., 2014).

For large-scale processes, the production of biopharmaceuticals from wild strains is normally avoided, mainly due to low yield. The use of recombinant DNA technology has been explored to increase production yield in enzyme processes (Adrio and Demain, 2010). High secretors and host strains of bacteria (e.g., *E. coli, Bacillus* and lactic acid bacteria), filamentous fungi (e.g., *Aspergillus*) and yeasts (e.g., *Pichia pastoris*) are used for the homologous and heterologous expression of recombinant enzymes (Goswami et al., 2015). Among them, bacterial hosts (e.g., *E. coli*) are most commonly used for ASNase production, since they can quickly and easily overexpress recombinant proteins (Ferrara et al., 2006; Liu et al., 2013; Costa et al., 2016).

Native *E. coli* ASNases, such as Elspar® (Merck, 2000) and the PEGylated form pegaspargase, Oncaspar® (European Medicines Agency, 2016) are produced industrially for medical application. The only recombinant ASNase available on the market so far, Spectrila®, is produced in *E. coli* as a host cell (European Medicines Agency, 2015). *D. chrysanthemi (E. chrysanthemi*) is also used as a host cell for producing the native ASNase, crisantaspase (Medicines Evaluation Board, 2015). Different strategies for protein engineering targeting the improvement of the ASNase therapeutic use are described in a latter section.

#### Media Composition and Cultivation Strategies

In addition to the microbial species used, ASNase production yields depend on the cultivation conditions. Thus, the identification of optimal culture media composition, temperature, pH, oxygen levels and others cultivation factors is of paramount importance. Several media compositions have been tested for ASNase production. In the case of wild type microorganisms, carbon and nitrogen sources have been reported as the most influencing factors, as reported in recent reviews (Kumar and Sobha, 2012; Cachumba et al., 2016; Lopes et al., 2017). Both submerged and solid state processes have been reported in the literature (Ashok and Kumar, 2017; Meghavarnam and Janakiraman, 2017), the later mainly for filamentous fungi. Nonetheless, only submerged cultivation is used for industrial production of ASNase for pharmaceutical use and ASNase from filamentous fungi is exclusively used in the food industry (Xu et al., 2016).

Another manufacturing alternative that has been studied to improve ASNase is the use of chemically defined culture media. According to Macauley-Patrick et al. (2005), in order to produce large quantities of heterologous proteins, the use of defined media is required so that the physicochemical environment can be manipulated and the protein expression maximized. It provides robustness, avoids variations caused by complex components and favors the downstream steps. Walsh (2010) reported that the use of a chemically defined culture medium improves the safety of biological drug production. Defined culture media were used by Macauley-Patrick et al. (2005) for ASNase production by recombinant *Pichia pastoris* and, according to the authors, the medium was considered ideal for large-scale production of heterologous proteins in bioreactors.

Alternatively to traditional batch cultivation, fed-batch is a strategy used to enhance ASNase production (Goswami et al., 2015), for both native and recombinant strains, since early works reported that ASNase synthesis can be catabolically repressed, i.e., it can be inhibited by glucose (Heinemann and Howard, 1969; Peterson and Ciegler, 1972). A study of continuous cultivation for ASNase production by *Erwinia aroideae* was carried out by Liu and Zajic (1973) and lower enzyme yields were obtained compared to batch cultivation, except when the process was conducted at a dilution rate of 0.1 h^−1^. Currently, no industrial ASNase production process is carried out in continuous mode.

Using fed-batch cultivation, successful results have been reported in the literature. Besides the productivity improvement, fed-batch cultivation allows the reduction of toxic by-product formation and simplifies the downstream processing, lowering overall production costs and reducing the technical effort required (Johnston et al., 2002). Goswami et al. (2015) achieved about 4-fold biomass (7.32 g.L^−1^ dry cell weight) and recombinant L-asparaginase II production (95.85 U.L^−1^) increase, using fed-batch compared to the batch process. Kumar et al. (2017) evaluated the production and productivity of a glutaminase-free ASNase from *Pectobacterium carotovorum* MTCC 1,428 both in batch and fed-batch process. In the batch process 17,97 U.L^−1^ and 1497.50 U.L^−1^.h^−1^ were achieved, while in the fed-batch process 38.8 U.L^−1^ and 1615.8 U.L^−1^.h^−1^ for the production and productivity, respectively, were achieved, corresponding to an increase of 115.8% in ASNase production and 7.9% in enzyme productivity (Kumar et al., 2017). The improved results of fed-batch for productivity justify its industrial use. Additionally, in a fed-batch process high-density-cell cultivation (HDC) can be reached, which increases the volumetric yield. Ferrara et al. (2006) reported the production of ASNase by recombinant *P. pastoris* in HDC in a 2 L bioreactor and the volumetric yield obtained was 85,600 U.L^−1^, global volumetric productivity was 1,083 U.L^−1^.h^−1^ (Ferrara et al., 2006).

The combination of recombinant DNA technology and HDC in the fed-batch process, allows enzyme production in much larger quantities than those obtained in traditional processes (Nakagawa et al., 1995; Roth et al., 2013) and enabled the reduction of the culture volume from 20,000 to 300 L in the Medac's Spectrila® production process (European Medicines Agency, 2015; Wacker Biotech, 2016). The application of this technology to the production of ASNase with the optimization of the cultivation process and an effective purification strategy increased the productivity, the yield and the quality of the ASNase produced, reducing the overall production costs while contributing to the technical-economic feasibility of the process.

#### Cultivation Optimization and Control

Before scale-up, the optimization of process variables is required. Statistical tools are employed to determine the effect of cultivation parameters on the process performance and outputs and to suggest/implement effective improvements (Agarwal et al., 2011). Statistical design of experiments (DoE) enables the study of several parameters at the same time, as well as of their interactions, from a minimum number of experiments. DoE can, therefore, help to understand the relation between process parameters and the final product quality profile (ICH, 2009). The screening of the major variables affecting the process can be carried out using methods such as the Plackett–Burman experimental design or the Taguchi's method (Placket and Brurman, 1946; Rao et al., 2008). Afterwards, in order to find optimal operating conditions, central composite design and the response surface methodology can be used (Managamuri et al., 2017). Other methods, such as model-based optimization are also popular (Zuo et al., 2014). Mechanistic and black box models (e.g., artificial neural network) can be used in conjunction with global, local or multiobjective optimization techniques, both deterministic (i.e., gradient-based) and non-deterministic (e.g., genetic algorithms) (Benyahia et al., 2010).

El-Naggar et al. (2015) evaluated fifteen variables in glutaminase-free ASNase production by *Streptomyces olivaceu*s NEAE-119 using the Plackett–Burman experimental design. Among the variables studied (temperature, pH, incubation time, inoculum size, inoculum age, agitation speed, dextrose, starch, L-asparagine, KNO_3_, yeast extract, K_2_HPO_4_, MgSO_4_·7H_2_O, NaCl, and FeSO_4_·7H_2_O), temperature, inoculum age and agitation speed were the most significant independent variables affecting enzyme production and were, therefore, targeted for optimization using the response surface methodology (El-Naggar et al., 2015). During the Plackett–Burman design experiments (20 runs), the maximum and minimum ASNase activity were 49.874 and 5.181 U.mL^−1^, respectively; after optimization a maximum of 70.46 U.mL^−1^ was achieved, and the model was validated with a high degree of accuracy (97.35%). The results obtained by El-Naggar et al. (2015) highlight the improvements that can be obtained by process optimization.

In the spirit of QbD, another important point that must be considered in bioprocess design is process control, which ensures a consistent quality of the final product (ICH, 2009). In traditional bioprocesses, real time control considers mainly the temperature, pH and dissolved oxygen. However, with the help of Process Analytical Tools (FDA, 2004a), other process parameters such as cell viability, cell density, substrate, product and by-product concentrations, dissolved carbon dioxide and other biomarkers can now be measured in real time. These Process Analytical Tools deliver crucial real time information about the process evolution and impacts of process perturbations, which in turn help monitor cell line variability and enable the implementation of advanced control strategies that can directly affect critical process parameters and critical quality attributes (Craven and Becken, 2015; Lakerveld et al., 2015).

### Downstream

Downstream processes present a large number of potentially critical process parameters that show interactions across unit operations and have to be investigated before scale-up (Meitz et al., 2014). Since 60–80% of the total production costs of biopharmaceuticals is usually associated with downstream processing, it has become crucial to investigate how to replace traditional methods with efficient and cost-effective alternative techniques for recovery and purification of drugs, decreasing the number of downstream unit operations (Buyel and Fischer, 2014; Tundisi et al., 2017). The integration of downstream unit operations is widely used for ASNase purification, through low resolution purification steps, i.e., fractional precipitation, aqueous biphasic systems, centrifugation and membrane-based purification (dialysis) (Zhu et al., 2007; Mahajan et al., 2014; El-Naggar et al., 2016) and high resolution purification steps, i.e., chromatographic processes.

#### Low Resolution Techniques

The first step of the ASNase downstream process is the drug release from the intracellular media. Most microbial ASNases are intracellular, few microorganisms are able to secrete it outside the cell (Amena et al., 2010), in these cases, the consequent ASNase purification will be harder due to the vast number of biomolecules released from intracellular media. Depending on the producer microorganism, the enzyme might be transported to the periplasmic space, as, for example, in the case of *E. coli* type-II ASNase, facilitating the consequent purification steps, since only the periplasmic proteins are released depending on the type and mode of operation of the disruption cell methods applied (Harms et al., 1991; El-Naggar et al., 2016). Costa-Silva et al. (2018) evaluated some disruption methods for ASNase release (produced by bacteria, yeast and filamentous fungus) using mechanical, chemical and physical methods. Mechanical methods were the most effective for all microbial cells used, including sonication and glass bead stirring. However, mechanical disintegration methods cause temperature increase (thereby resulting in ASNase denaturation) and, in general, this disruption mechanism co-releases byproducts such as nucleic acids, cell fragments and others proteins with the intended product. Therefore, extracellular ASNase is considered more advantageous than the intracellular type for the downstream process, since the enzyme can accumulate in the culture broth under normal conditions, simplifying the extraction process. However, periplasmic ASNase production facilitates protein folding, as there is a more favorable redox potential in the periplasmic space (El-Naggar et al., 2016). In laboratory scale, osmotic shock is used to release ASNase from the periplasmic space (Harms et al., 1991), in the industrial scale, this technology is, however, not easy to be implemented. In the reports of the periplasmic ASNase production available in the market, this step is not well described (European Medicines Agency, 2015, 2016).

Therefore, alternative methods have been developed for application on an industrial scale that can lead to high purification factors and purities, compromising the use of integrated purification steps. For instance, Wagner et al. (1992) patented a method of extraction of periplasmic ASNase produced by *E. coli* through cell flocculation and centrifugation, followed by resuspension in water and precipitation by a water-miscible solvent. With the addition of an organic base, the extracted cells, nucleic acids and ballast proteins are precipitated and removed by centrifugation. Finally, ASNase is precipitated from the cell-free solution with acetone.

Yu et al. (2018) studied the purification of recombinant ASNase from *E. coli* using crystallization by solvent freezing technology (SFO) with methanol, 2-methyl-2,4-pentanediol (MPD), polyethylene glycol (PEG) 6,000 and ethanol as precipitating agents. Initially, the biomass was disintegrated via high pressure homogenization (1,000 bar). Cold acetone was then injected followed by centrifugation. The pellet, referred as the crude extract, was used for further purification by crystallization. Specific activity increased from 27 to 135 U.mg^−1^ after purification by crystallization. However, SDS-PAGE showed that, in addition to an ASNase band at 35 kDa, there were still other bands, indicating protein contamination in the crystals of ASNase II demonstrating, therefore, the need to associate other techniques to crystallization by SFO to obtain pure ASNase.

Santos et al. (2018) proposed the purification of periplasmic ASNase from *E. coli* using precipitation with ammonium sulfate followed by a separation with a polymer/salt based aqueous biphasic system (ABS). The novelty of this work was the important role of ionic liquids acting as adjuvants, enhancing the purification factor of ASNase. The results revealed preferential partitioning of ASNase to the polymer rich-phase of the system owing to the hydrophobic effect resulting from van der Waals interactions between enzyme and polymer. Low amounts of ionic liquids in the ABS were sufficient to achieve about 90% recovery of ASNase with high purity.

#### High Resolution Techniques

Despite major advances in the development of low resolution separation methods such as precipitation, aqueous biphasic systems and crystallization, chromatography-based techniques continue to be the backbone of the biopharmaceutical industry (Rathore et al., 2018). Chromatography is still widely preferred thanks to its scalability, robustness, selectivity, high clearance of impurities and most importantly easy validation compared to other purification processes (Soares et al., 2012). It includes different techniques with their own characteristics such as hydrophobic interaction (HIC), ion exchange (IEX) and gel filtration (SEC) chromatography. These separations are based on biomolecules hydrophobicity, charge and size, respectively. As mentioned before, in order to consolidate a technological process and turn it economically viable, the number of intrinsic steps must be reduced. The best efficiency can be achieved through the synergism between different unit operations involving techniques that can be scaled in an industrial context (Cortez and Pessoa, 1999; Dux et al., 2006).

Mangamuri et al. (2016) studied the effect of an integrated downstream process to purify an extracellular ASNase from *Pseudonocardia endophytica* VUK-10. By employing a 3-step downstream protocol involving ammonium sulfate precipitation, gel filtration chromatography and ion exchange chromatography, the specific activity of ASNase increased from 7.31 to 702.04 U.mg^−1^ with 61% yield.

Moreover, for recombinant His-tagged ASNases the immobilized metal ion affinity chromatography (IMAC), using a ${\text{Ni}}_{2}^{+}$-charged resin, is highly recommended. The use of IMAC proved to be an efficient tool in the purification of recombinant ASNase I of *S. cerevisiae*, resulting in high recoveries (40.50 ± 0.01%) and a purification factor of 17-fold (Santos et al., 2017).

Regarding the downstream processing of two biopharmaceutical ASNases currently available on the market: Oncaspar® and Erwinase®, integrated purification platforms are used. For Oncaspar®, the purification includes hydrophobic interaction chromatography, anion and cation exchange chromatography as well as clearance steps to effectively deplete product and process related impurities. For the polishing steps, the active substance is dispensed into sterile containers and subjected to testing and release. Meanwhile, for Erwinase® the extracts are pooled and processed through a series of column chromatography and other protein purification steps to yield a drug substance batch (Medicines Evaluation Board, 2015; European Medicines Agency, 2016).

A challenge to overcome in the use of therapeutic proteins refers to soluble or insoluble aggregates which, when administered, may trigger immunogenic reactions (Cromwell et al., 2006; Eon-Duval et al., 2012). A major breakthrough in that direction was achieved in the development of the recombinant ASNase Spectrila® (Medac GmbH, Hamburg, Germany), which succeeded in reducing the aggregate content in the final product from 20.5 to < 0.3% (European Medicines Agency, 2015).

## Protein Engineering for Improvement of L-Asparaginase Therapeutic Use

In accordance to QbD tenets, innumerable techniques have been proposed to overcome ASNase treatment downsides and, therefore, to develop biobetter ASNases. Protein engineering using bioinformatics analysis, molecular dynamics, docking and site-directed mutagenesis is among the most sophisticated techniques (Mundaganur et al., 2014; Nguyen et al., 2016; Ardalan et al., 2018). Table 1 summarizes the amino acid alterations by some of the techniques cited above aiming at improving ASNase properties.

**Table 1:** Summary of the amino acid substitutions on several L-asparaginases and the results achieved.

**Source**	Mutations^*^	**Results achieved**	**Reference**
	N248A	0.2% glutaminase activity and 12% L-asparaginase activity	Derst et al., 2000
	R195A/K196A/H197A	Reduction in antigenicity	Jianhua et al., 2006
*E. coli* (Eca II)	N178P	Retention of 90% L-asparaginase activity at 50°C (wild-type 71%)	Li et al., 2007
	N24G	AEP resistant-Retention of 45% L-asparaginase activity	Patel et al., 2009
	N24A/R195S	50% glutaminase activity and ≅100% L-asparaginase activity	Offman et al., 2011
	N24A/Y250L	≅0% glutaminase activity and ≅72% L-asparaginase activity	
	Y176S	Increase of V_max_/K_M_ for L-aspartic acid beta-hydroxamate	Mehta et al., 2014
	W66Y	Induced significantly more apoptosis in lymphocytes from ALL patients	
	Y176F	Glutaminase activity reduction and ≅100% L-asparaginase acctivity	
	Y176S	Glutaminase actvity reduction and ≅100% L-asparaginase activity	
	K288S/Y176F	Glutaminase activity reduction and ≅100% L-asparaginase activity	
	K288S/Y176F	10-fold less immunogenic	
	K139A	Retention of 65% L-asparaginase activity at 65°C (wild-type 40%)	Vidya et al., 2014
	L207A	Retention of 57% L-asparaginase activity at 65°C (wild-type 40%)	
	Y176F	Increase of V_max_/K_M_ for L-aspartic acid beta-hydroxamate	Verma et al., 2014
	Q59L	0% glutaminase activity and 80% L-asparaginase activity	Chan et al., 2014
	N24S	Improved thermal stability and proteases resistant	Maggi et al., 2017
	L23G/K129L/S263C/R291F	Non-toxic, more stability and longer half life	Mahboobi et al., 2017
	V27T	Glutaminase activity reduction and more stable	Ardalan et al., 2018
*Erwinia carotovora*	R206H	Enhanced resistance to trypsin degradation and higher thermal stability	Kotzia et al., 2007
*Erwinia chrysanthemi*	N133V	Higher thermal stability	Kotzia and Labrou, 2009
*Pectobacterium carotovorum*	N96A	Decreased glutaminase activity (30%) and increased asparaginase activity (40%)	Ln et al., 2011
*Pyrococcus furiosus*	K274E	Resistant to proteolytic digestion and no displayed glutaminase activity	Bansal et al., 2012
*Rhodospirillum rubrum*	D60K, F61L	Improvement of kinetic parameters and enzyme stabilization	Pokrovskaya et al., 2015
*Helicobacter pylori*	T16D	Deplete the enzyme of both its catalytic activities	Maggi et al., 2017
	T95E	Deplete the enzyme of both its catalytic activities	
	Q63E	Halved glutaminase efficiency	
	M121C/T169M	Without L-glutamine hydrolysis	
*Saccharomyces cerevisiae*	T64A, Y78A, T141A, K215A	99.9% loss of activity	Costa et al., 2016

* *Mutation: Y176S Y = Original amino acid; 176 = Residue position on the ASNase amino acid sequence; S = New introduced amino acid*.

### Reducing Glutaminase Activity

Although the glutaminase activity of ASNase is important for its activity against asparagine synthetase positive cancer cells (Chan et al., 2014), most of the side effects of ASNase treatment previously mentioned have been attributed to it (Warrell et al., 1982; Kafkewitz and Bendich, 1983). Therefore, from a quality perspective, a reduction on glutaminase activity is desirable.

Commercial ASNase used in the lymphoblastic leukemia and lymphosarcoma treatment are from bacterial sources and are not “glutaminase-free”. The *E. coli* (EcA II) and *E. chrysanthemi* (ErA) ASNases hydrolyzes L-glutamine up to 9% of total hydrolysis activity (high preference for L-Asn over L-Gln). Therefore, innumerable approaches to obtain ASNase with lower glutaminase activity have been investigated, such as bioprospecting other microbial sources or the modification of commercial ASNases by site-directed mutagenesis (Loureiro et al., 2012; Ardalan et al., 2018). Amino acids essential for biocatalysis in most cases were not altered since they are obligatory for effective ASNase binding to asparagine. Alternatively, several amino acids that surround the active site without directly interacting with the substrate, seemed to be better residues for targeted substrate specificity alteration (Offman et al., 2011; Mehta et al., 2014).

Derst et al. (2000) demonstrated that in native Eca II the Asn 248 is involved in hydrogen bonding that influences substrate binding. By replacing this Asn by Ala, they observed a reduction in glutaminase activity (Derst et al., 2000). Nevertheless, even though this residue does not belong to the catalytic site, this modification also substantially decreased the ASNase activity (about 12% of ASNase activity retention). Offman et al. (2011) used site-directed mutagenesis and created the double mutants N24A/Y250L, which almost eliminated glutaminase activity and retained ≅72% of ASNase activity. L-glutamine is larger than L-asparagine and this feature can be exploited to change the enzyme activity. The ASNase active-site is partially located at the monomer-monomer interface, and, consequently, changes in the tetramer compactness and/or active-site cavity volume will probably affect the glutaminase activity. The double mutant N24A/Y250L resulted in higher tetramer compactness with a smaller active-site cavity volume than the native enzyme, justifying the decrease of glutaminase activity (Offman et al., 2011).

Other studies investigated additional site-directed mutagenesis promoting a substantial decrease of glutaminase activity (Chan et al., 2014; Mehta et al., 2014). Recently, Ardalan et al. (2018) applied molecular docking studies, quantum mechanics and molecular dynamics simulations to engineer the *E. coli* ASNase (EcA II) in order to obtain a mutant without glutaminase activity (Ardalan et al., 2018). They excluded residues present in the active-site or which are responsible for substrate binding and selected the residue V27 based on the binding energy (binding energy to L-asparagine: −0.182 kcal.mol^−1^ and binding energy to L-glutamine: −0.4758 kcal.mol^−1^). The V27T (nonpolar to polar amino acid) mutant showed higher glutaminase free binding energy (from −20.763 to 1.360 kcal.mol^−1^) and higher stability than the native enzyme, while retaining ASNase activity. The number of hydrogen-bonds between ASNase and glutamine were reduced, lowering the interaction of the substrate with the active-site (Ardalan et al., 2018). All the studies that completely eliminated the glutaminase activity affected to some extent the ASNase activity due the fragility of the network in the active-site.

### Increasing *in vivo* Stability

Commercial ASNases used in lymphoblastic leukemia treatment are characterized by low *in vivo* stability resulting in the need of several administrations. Therefore, a desirable quality for potential biobetter candidates would be a longer half-life, which can be achieved by designing protease-resistant ASNases.

Asparagine endopeptidase (AEP) and cathepsin B (CTSB) are two human lysosomal proteases which contribute to ASNase short half-life (Patel et al., 2009) and site-directed mutagenesis has been used to create protease-resistant ASNase. Patel et al. (2009) identified the residue N24 as the primary cleavage site for AEP and proposed the change of this residue to G24. ASNase N24G mutant was resistant to AEP cleavage but was substantially less active (relative activity 45%). This residue (N24) is not directly involved in catalysis but it is responsible for active-site stabilization (Maggi et al., 2017).

Using molecular dynamics simulations the N24S mutation was proposed and a new ASNase showing resistance to proteases derived from leukemia cells was obtained, which retained all original enzymatic activity (Maggi et al., 2017). The improved biochemical characteristic of N24S provides a potential alternative to improving outcome in childhood ALL treatment.

### Reducing Immunogenicity

The reduction of immunogenicity is fundamental for the development of biobetter ASNases, given that the development of an immune response reduces treatment efficacy and may lead to potentially dangerous reactions. Therefore, from the QbD perspective, a reduction in immunogenicity would result in an improved quality profile. Studies involving bioinformatics analysis, antigenic peptide prediction, prediction of B-cell epitopes and site-directed mutagenesis were carried out and revealed numerous B-cell epitopes on the surface of *E. coli* ASNase II which are accountable for immunogenicity (Jianhua et al., 2006; Mehta et al., 2014; Mahboobi et al., 2017). To overcome this limitation, several ASNase variants have been created. Alanine-scanning mutagenesis was applied to identify the residues that are important to the antigenicity and a sequence change from 195RKH197 to 195AAA197 was proposed, resulting in reduced enzyme antigenicity (Jianhua et al., 2006). Mahboobi et al. (2017) used several bioinformatics software to improve ASNase pharmaceutical profile and obtained one enzyme variant with four mutations (L23G, K129L, S263C, and R291F) presenting lower toxicity, higher stability and increased half-life. Other site-directed mutations performed to different ASNases are summarized in Table 1.

### Perspectives for the Future of L-Asparaginase Engineering

Rational protein engineering is a very promising approach to obtain new ASNase proteoforms with improved quality profile, such as: reduced or eliminated glutaminase activity, resistance to proteases, long-term stability, improved thermal stability and decreased immunogenicity. These desirable features of new ASNase variants could significantly improve the enzyme therapy of acute lymphoblastic leukemia in the future.

Unfortunately, a new ASNase containing all these characteristics has not yet been developed. Using genetic engineering, changes in residues not involved in the catalytic site, as the introduction of recombinant cysteine residues in the protein surface, are possible, which could, for example, improve ASNase PEGylation, and prevent dissociation-induced loss of activity (Ramirez-paz et al., 2018). Another strategy with great potential is the use of expression systems capable of expressing glycosylation patterns similar to those of mammals or even human-like (Sajitha et al., 2015; Nadeem et al., 2018). The glycosylation can improve the enzyme's pharmacokinetics, solubility, distribution, serum half-life, effector function, and binding to receptors, and can, therefore, be used to reduce many of the treatment side effects (Nadeem et al., 2018).

Several techniques can be used to predict the best protein engineering strategy and the majority of them are based on bioinformatic tools for modeling and modifying the ASNase properties such as: genetic algorithm, structure-based multiple sequence alignment, crystallographic structure analysis and molecular dynamics simulations, three-dimensional structure modeling, molecular docking studies, circular dichroism, measurements solvent accessibility of the tetramer and protein internal dynamics, kinetic competition analysis and energy minimization (kinetics of Asn and Gln catabolism), binding free energy computation, density functional theory (DFT) and innumerable prediction studies (antigenic and allergenic peptide prediction, conformational stability prediction, hydrogen-bonded turn structures prediction). All of these emerging approaches involving bioinformatics tools and functional/structural analysis should be used to exploit the thoroughgoing potential of this biopharmaceutical.

Seen as the future of genetic engineering and gene therapy, the CRISPR/Cas9 technique has gained much prominence in the last years. However, to the best of our knowledge, no applications of it on the engineering of ASNase have been reported in the scientific literature. Interestingly, there have been some reported instances of its use on the elucidation of the genetic markers responsible for ASNase-therapy resistance in certain ALL cell lineages, which has led to promising new treatment proposals (Butler et al., 2017; Ding et al., 2017; Hinze et al., 2018; Montaño et al., 2018).

## L-Asparaginase Formulation

ASNase pharmaceutical dosage in the market (intravenous/intramuscular solution) is available in four different formulations, as described in Table 2. In these pharmaceutical formulations, excipients have been used to stabilize the enzyme structure and reduce protein aggregation. Salts (phosphate buffer), sugars and polyols (sucrose, glucose and mannitol) have been reported to ensure ASNase stability during freeze-drying process and to enhance its shelf-life (Ohtake et al., 2011).

**Table 2:** Current biopharmaceutical formulations for L-Asparaginase (ASNase).

**Approved Biopharmaceuticals**	**Pharmaceutical form/aspect**	**Route of administration**	**Composition**	**References**
ASNase amidohydrolase	Lyophilized white crystalline powder, water soluble (225 IU/mg)	Intravenous/intramuscular	Mannitol (80 mg in 10,000 IU of the enzyme)	Merck, 2000
ASNase from *Dickeya chrysanthemi (Erwinia chrysanthemi*) (Erwinase®)	Lyophilized white powder, water soluble (10,000 Units)	Intravenous/intramuscular	5 mg glucose per bottle < 1 mmol sodium (23 mg) per dose, i.e., essentially sodium-free	Medicines Evaluation Board, 2015
Recombinant ASNase from *Escherichia coli (Spectrila*®)	Lyophilized white powder, water soluble (10,000 Units)	Intravenous	Sucrose	European Medicines Agency, 2015
Pegylated ASNase amidohydrolase (Oncaspar®)	Clear, colorless, preservative-free, isotonic sterile solution (3,750 IU/5 mL)	Intravenous/intramuscular	Phosphate buffer (1.20 mg monobasic sodium phosphate, 5.58 mg dibasic sodium phosphate, and 8.50 mg sodium chloride per mL of water for injection)	European Medicines Agency, 2016

Sugars are not effective against shear and interfacial stresses, but these molecules are suitable to protect against dehydration, freezing and thermal stress during the lyophilization of Elspar®, Erwinase®, and Spectrila®. Theories on vitrification and water replacement are proposed to explain the mechanism by which sugars are able to stabilize ASNase in the solid state. According to the vitrification theory, the protein is immobilized in a rigid, amorphous glassy sugar matrix, preventing molecular mobility (Ohtake et al., 2011). Otherwise, according to the water replacement theory, hydrogen bonds between the protein and the hydroxyl groups of the sugars are formed upon drying replacing hydrogen bonds between water and the protein, contributing to protein stabilization (Wlodarczyk et al., 2018).

Salts are present in the current PEGylated formulation Oncaspar®, classically in the form of buffers, and may improve the stability of proteins in solution by increasing the chemical potential of the system. However, they are not expected to confer any stability to proteins in the dried state due to their crystallization (Ohtake et al., 2011).

### Immobilization

It is noteworthy that over more than 40 years since the approval of the first ASNase formulation (Elspar®) by the Food and Drugs Administration (FDA) in 1978, the only technological innovation to emerge was the PEGylated ASNase (Oncaspar®) in 1994. One of the most complex problems of the ASNase treatment is the silent inactivation of the enzyme due to the expression of anti-asparaginase antibodies (Zalewska-Szewczyk et al., 2007) as well as due to proteolysis by lysosomal proteases present in leukemic cells (Patel et al., 2009). An optimized drug delivery system is, therefore, essential for an ASNase product design within the QbD framework.

ASNase immobilization in nanostructured materials, i.e., the enzyme confinement in different types of substrates/supports, would be an alternative to overcome the drug drawbacks since it protects enzymes from the action of proteases and expands the catalytic half-life *in vivo* (Bosio et al., 2016). Three main forms of enzymatic immobilization have been described in the literature: adsorption, covalent bonds and encapsulation. The adsorption results from the hydrophobic interactions between the enzyme and the carrier including van der Waals forces, ionic interactions and hydrogen bonds. These interactions are rather weak and there are no changes in the native structure of the enzymes (Datta et al., 2012; Jesionowski et al., 2014). Covalent bonds occur in the amino acid chain itself in residues such as arginine, lysine, aspartic acid and histidine. Encapsulation is a distinct method of enzyme immobilization by inclusion, which is mainly based on the principle of immobilization without attachment to the carrier material (Rother and Nidetzky, 2014).

Several materials were already investigated for ASNase immobilization, such as albumin, poly(DL-alanine) peptides, dextran, dextran sulfate, chitosan, N,O-carboxymethyl chitosan colominic acid, glyoxyl-agarose, agarose-glutaraldehyde, fructose, levan, inulin, alginate, gelatin, silk fibroin, silk sericin, fatty acids, BSA, phospholipid DPPC PEG, PEG-albumin chemical, calcium alginate-gelatin, cross-linking, PEG-chitosan and glycol-chitosan conjugation (Ulu and Ates, 2017).

Vina et al. (2001) reported the immobilization of *Erwinia chrysanthemi* ASNase on the polysaccharide *levan* through an oxidation reaction with potassium periodate followed by reductive alkylation. The reaction occurs in Lys residues and N-terminal amino groups of ASNase at pH 9-9.2, in which the amine groups are preferably deprotonated. According to the authors, an increase in the apparent K_M_ of the enzyme was observed with a small decrease in enzyme activity. The range of pH stability was, on the other hand, broadened. Immobilized ASNase showed higher thermal stability and 1-month stability over storage in aqueous solutions compared to the native enzyme.

Covalent immobilization of ASNase was also performed on various activated agarose supports followed by crosslinking with high molecular weight dextran aldehydes. Enzyme activity decreased with immobilization, however with a significant increase in half-life (Balcao et al., 2001).

Immobilization in sericin (silk protein) through glutaraldehyde crosslinking has also been investigated resulting in higher thermal stability and resistance to degradation by trypsin. Furthermore, the apparent K_M_ of sericin-conjugates was 8 times lower than that of non-immobilized ASNase (Zhang et al., 2004). In another work, ASNase immobilized on poly(methyl methacrylate) with starch resulted in a decrease in the apparent K_M_ of around 8-fold compared to the value of the non-immobilized enzyme. Moreover, after 1-month storage period at 4°C the immobilized ASNase retained 60% of activity (Ulu et al., 2016).

### Nanoencapsulation

In 2014, the FDA published a *Guidance for Industry Considering Whether an FDA-Regulated Product Involves the Application of Nanotechnolog*y and, as a result, some FDA-approved nanomedicines are currently available: antibody–drug conjugates, liposome-based delivery platforms, and albumin-bound nanoparticles, all with enhanced permeation and retention (EPR) effect for extravasation from the circulation and accumulation at the tumor site. However, for LLA, a non-solid cancer of the blood and bone marrow, there is no requirement of EPR effect. In this case, an efficacious nanobiotechnology-based biopharmaceutical demands, instead, long-circulating nanoparticles (Blanco et al., 2015).

ASNase encapsulation into nanoparticles and liposomes has been described in the literature; however, there are no nanoformulations currently in use in the clinical practice. The development of nanotechnology-based ASNases is still a challenging task and some key aspects must be taken into account. First, the starting material for the nanocarriers such as polymers, lipids and surfactants should be rationally chosen considering that a prerequisite for most of the pharmaceutical products is biodegradability, to ensure later elimination from the body (Keck and Müller, 2013). Moreover, the material should also be sterilizable and, particularly for ASNase (which must be refrigerated for hospital usage), must be stable at lower temperatures.

Another major issue associated with the ASNase nanotechnological development is the scale-up process and its inherent challenges (Paliwal et al., 2014). Several methods are available at laboratory scale to produce nanostructures for protein drug delivery, but scale up demands high efficiency, a fast and continuous process and adequate methods for thermolabile proteins like ASNase (e.g., cold homogenization process) (Paliwal et al., 2014; Pachioni-Vasconcelos et al., 2016; Apolinário et al., 2018). Furthermore, solvent free processes and reduced shear stress are preferable to avoid ASNase denaturation and/or aggregation. Lack of control of nanocarrier size and polydispersity are two common pitfalls for some of the production methods employed in small scale such as film hydration (Apolinário et al., 2018). To solve this problem, size exclusion chromatography can be added as a nanostructure purification step (Wang et al., 2010).

Control of nanosystems polydispersity is essential. This parameter describes the heterogeneity of particles regarding to size or mass and, if subject to even small variations, could result in dramatic changes in the nanocarriers properties such as biocompatibility, toxicity and *in vivo* distribution (Wicki et al., 2015). In addition, nanocarriers below 100 nm can be internalized by any cell by endocytosis (Keck and Müller, 2013) and to avoid fast clearance by the kidneys, nanocarriers should be larger than 8 nm (Dawidczyk et al., 2014). Therefore, nanosystems should be characterized on a batch-to-batch basis. Table 3 shows some examples of nanoencapsulation strategies already studied for ASNase, including the characterization techniques employed. In this Table, ASNase encapsulation efficiency (EE%) was expressed as the percentage ratio between the enzyme concentration in the nanocarriers and the total enzyme used in the production process.

**Table 3:** Nanoencapsulation strategies for L-Asparaginase (ASNase).

**Nanocarrier**	**Material**	**Technique**	**Characterization**	**Encapsulation efficiency or activity recovery**	**References**
Nanoparticles containing PEG-ASNase	Poly (lactide-co-glycolide) nanoparticles 50:50 with molecular mass of 10 kDa	Double emulsification	Size and morphology by Dynamic light scattering (DLS) and scanning electronic microscopy (SEM)	77.88% for free ASNase and 65.1% for pegylated enzyme	Suri Vasudev et al., 2011
Nanoparticles	Chitosan-tripolyphosphate	Ionotropic gelation	Size and morphology by Transmission electronic microscopy (TEM) and DLS	59.1–70.8%	Bahreini et al., 2014
Nanoparticles	Poly (lactide-co-glycolide) nanoparticles 50:50 with molecular mass of 30 kDa	Double emulsification	Size and morphology by TEM	5%	Manuela Gaspar et al., 1998
Nanoparticles	Poli-(3-hydroxybutyrate-co-3-hydroxyvalerate	Double emulsification	Size and morphology by SEM	23.7% for free ASNase and 27.9% for pegylated enzyme	Baran et al., 2002
Nanoparticles	Poly (lactide-co-glycolide) nanoparticles 50:50	Double emulsification	Size distribution were examined by laser diffraction	26–70%	Wolf et al., 2003
Microparticles	Silk sericin protein with different molecular mass from 50 to 200kDa	Crosslinking with glutaraldehyde	Size distribution were examined by laser diffraction	62.5% of the original activity of the ASNase	Zhang et al., 2004
Hollow nanospheres	Alginate-graft-poly (ethylene glycol) (Alg-g-PEG) and a-cyclodextrin (a-CD)	Self-assembly	Size and morphology by TEM and DLS	37–80%	Ha et al., 2010
Magnetic nanoparticles	SiO_2_, Fe_3_O_4_, poly(2-vinyl-4,4-dimethylazlactone)	Formation in alkaline medium followed by washing with water until neutral pH	Size and morphology by TEM and DLS	107–318 amount of enzyme (μg.mg^−1^ nanoparticle)	Mu et al., 2014
Liposomes	Egg phosphatidylcholine, egg phosphatidylinositol, cholesterol and other lipids	Film hydration with or without extrusion	Size by TEM DLS	40% for extruded sample and 80% for non-extruded sample	Cruz and Gaspar, 1993
Liposomes	Phosphatidylcholine, cholesterol and other lipids with or without charge	Film hydration	Size and morphology by SEM and DLS, zeta potential	1.95% neutral lipids and 2.39% for positive lipids and 2.35% for negative ones	Anindita and Venkatesh, 2012
Liposomes	Soybean phospholipid and cholesterol	Reverse-phase evaporation method	Size and Morphology by TEM and DLS, zeta potential	66.47%	Wan et al., 2016
Polyion complex vesicles (PICsomes)	Polyethylene glycol and homoionomers	Electrostatic-interaction-mediated self-assembly in aqueous media	Size and morphology by DLS and Cryo-TEM	91% of the PICsomes were loaded with at least one molecule of ASNase	Sueyoshi et al., 2017
Red Blood Cells (RBC)	*E. coli* ASNase loaded into homologous RBC at a concentration of 50% and suspended in saline, adenine, glucose, mannitol	3-h automated process: I) the preservative solution is removed from the packed RBC by a washing step II) ASNase is and RBC are put together in the washed suspension, III) Dialysis of this mixture is against a hypotonic solution and resealed, IV) Purification of the product through a final washing step V) the preservatives are added	Concentration and activity of ASNase, extracellular hemoglobin, osmotic fragility	—	Bailly et al., 2011
Polymersomes	Poly (2-hydroxypropyl methacrylate)	Polymerization-induced self-assembly	Size and morphology by DLS and Cryo-TEM	9%	Blackman et al., 2018
Polymersomes	Poly (ethylene glycol)-poly (lactic acid)	Film Hydration	Size and morphology by DLS and TEM	5–20%	Apolinário et al., 2018

The ASNase release from the nanocarriers is also challenging since a balance is required to avoid fast clearance or late release. Nanosystems should be stable during the biological distribution and should not be altered under flow and at physiological temperature. Additionally, the nanocarriers should not signifficantly bind to blood components, which could lead to aggregation, nonspecific binding to the endothelium or uptake by the mononuclear phagocyte system (Dawidczyk et al., 2014). For the anti-leukemic function through asparagine depletion, ASNase must be available in the bloodstream, so a release stimuli-sensitive trigger is not necessary.

Recently, permeable polymersomes were developed as ASNase nanobioreactors. These polymersomes lead to L-asparagine depletion without ASNase release from the nanostructures into the bloodstream, reducing the enzyme proteolytic degradation and antibody recognition compared to free protein or PEGylated conjugates (Blackman et al., 2018). A red blood cell (RBC) erythrocyte encapsulated ASNase (GRASPA®) was also developed as a “cellular microbioreactor,” allowing intracellular depletion of L-asparagine over a longer period than the native form of the enzyme while using lower doses. This system is in phase-III clinical trial (Domenech et al., 2011). More than a microbioreactor, GRASPA® is an example of personalized drug delivery, since the RBC material is selected to be compatible with the patient immunologic and hematologic profile (European Medicines Agence, 2016).

### PEGylation

Protein PEGylation is an important technique for the development of biopharmaceuticals with an improved quality profile, as prioritized by QbD principles (Turecek et al., 2016). Although PEGylation was firstly described in the late 1970s by Franck Davis and his colleagues (Abuchowski et al., 1977; Hoffman, 2016), new frontiers for this technology are now emerging, through advances in PEGylation chemistry and extension to a plethora of novel protein-based biopharmaceuticals (Ryu et al., 2012; Ginn et al., 2014; Swierczewska et al., 2015). Protein PEGylation consists in the attachment of polyethylene glycol (PEG) - an FDA approved polymer-to a protein-based drug, aiming at improving circulation half-life by reducing the rate of glomerular filtration (Ginn et al., 2014; Kolate et al., 2014; Turecek et al., 2016).

**Figure 3:**
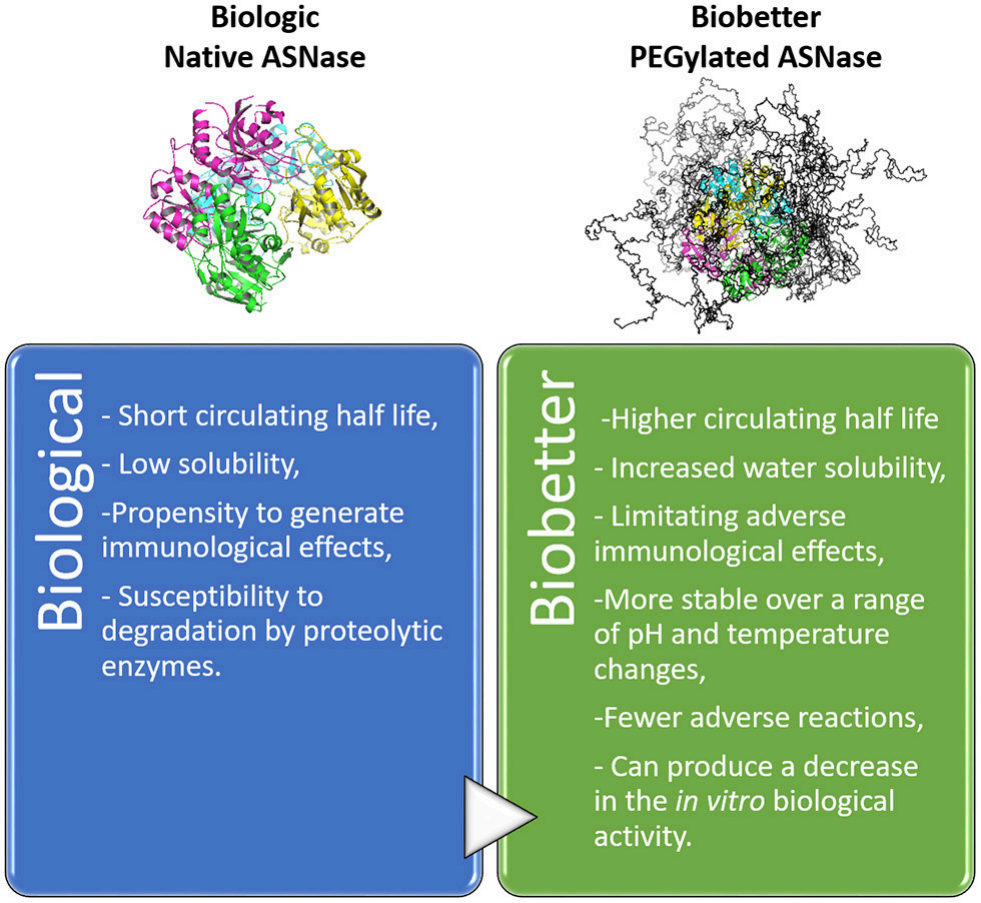
Influence of PEGylation as an engineering technique for biobetter drug development. Example: native ASNase and the respective biobetter–PEGylated ASNase (9 PEG chains of 10 KDa).

PEGylated proteins have arisen in the biopharmaceutical field as an endeavor to improve the clinical properties of protein-based biologics in terms of increasing circulation half-life without affecting the biological activity (Ginn et al., 2014; Turecek et al., 2016). Not only is the drug half-life boost witnessed an advantage of PEGylation, but also the enhancement of therapeutic efficacy of drugs, through several other beneficial effects, as described in Figure 3 (Harris and Chess, 2003; Ginn et al., 2014; Swierczewska et al., 2015; Turecek et al., 2016). PEGylation decreases protein aggregation by increasing its hydrophilicity, decreasing proteolytic degradation and recognition by anti-drug antibodies. However, PEGylation can negatively affect the *in vitro* activity of protein-based biologics. This effect can be offset in biological systems by the longer period of the drug circulation in blood vessels (Turecek et al., 2016).

Pegaspargase (Oncaspar®) was one of the first PEGylated biobetters approved by the FDA in 1994. Later on, in 2006, the FDA granted approval to pegaspargase (Oncaspar®) for the first-line treatment of patients with acute lymphoblastic leukemia (ALL) as a component of a multi-drug chemotherapy treatment (Dinndorf et al., 2007). This biobetter is obtained by widespread covalent bond of succinimidyl-succinate-activated 5 kDa methoxi-PEG to the protein amine groups, which drastically reduced the immunogenic activity and prolonged the bloodstream residence time. Since PEGylation is random, polydispersity is considerable in pegaspargase formulations (i.e., 69–82 molecules of methoxi-PEG 5 kDa attached to the protein) (Lopes et al., 2017).

PEGylation on amine groups is considered a first generation PEGylation process, since it refers to random attachments of PEG to lysine/N-terminal groups, resulting in a mixture of isomers with batch-to-batch variation, an undesirable characteristic from the QbD perspective (Zalipsky, 1995; Harris and Chess, 2003; Santos et al., 2018). Despite these limitations, Oncaspar® received regulatory approval and is still in use as a first-line treatment of ALL in some countries.

Pegaspargase has a longer half-life when compared to native enzymes (Avramis et al., 2002; Dinndorf et al., 2007) (5 and 10 times longer than *E. coli* and *E. chrysanthemi*) and lower levels of hypersensitivity (Ho et al., 1986; Keating et al., 1993; Panosyan et al., 2004). A major limitation of *E. coli* ASNase is hypersensitivity, reported in 15–73% of adults and children. In addition, a single injection of pegaspargase can be given instead of the inconvenient administration of multiple doses of native ASNase (Avramis et al., 2002; Douer et al., 2006). The lower immunogenic profile of pegaspargase was proven *in vivo*, since it was shown to reduce antibody formation in animal models compared with the native drug (Dinndorf et al., 2007; Lopes et al., 2017). The SS linker in the PEG derivative contains an ester group, which has limited stability *in vivo* due to hydrolysis by endogenous esterases, nevertheless PEGylated ASNase still exhibits a higher residence time in the bloodstream (Carter and Meyerhoff, 1985).

Emerging biobetters, such as PEGylated drugs, require pharmacoeconomic analyses in order to understand overall treatment costs compared to the originator biologics (Oderda, 2002; Sassi et al., 2015). Pharmacoeconomic analyses of native pegaspargase vs. *E. coli* ASNase are still scarce in the literature, nonetheless in the past years, some studies addressing this important issue were published (Rees, 1985; Peters et al., 1995; Kurre et al., 2002). A study from the Children's Cancer Group (CCG), designed to collect medical and nonmedical cost information, compared pegaspargase and the native *E. coli* ASNase treatment of patients with standard-risk ALL from payer and societal perspectives and showed that the costs of the two therapies were similar from the payer perspective, with pegaspargase costing 1.8% more than *E. coli* ASNase. Additionally, the fewer medical care visits and side effects with pegaspargase were found to counterbalance the supplementary cost (patients treated with pegaspargase incurred total medical and nonmedical costs of $13,261 during induction, compared to $14,989 for *E. coli* ASNase). In conclusion, pegaspargase should not be excluded from a treatment protocol solely because of its high acquisition costs, since the treatment costs are similar to *E. coli* ASNase (Rees, 1985).

### Current Trends in the Development of Novel Biobetter L-Asparaginases

Studies considering other kinds of bioconjugates have been reported aiming at the improvement of the ASNase quality profile (i.e., its pharmacokinetic and immunological properties) and the production of novel biobetter ASNases. Tabandeh and Aminlari (2009) investigated ASNase conjugation with oxidized inulin and improved pharmacokinetic and physicochemical characteristics were observed, such as resistance to trypsin digestion and higher thermal stability, longer half-life, better reusability after repeated freezing and wider optimum pH range than that of native ASNase. Furthermore, bioconjugation resulted in the decrease of antibody (IgG) titer and immunogenicity after repeated injections of rabbits when compared to the native ASNase. Zhang et al. (2005) prepared silk fibroin-ASNase bioconjugates that presented reduced immunogenicity and antigenicity, good residual activity (nearly 80%), increased thermal and storage stability, resistance to trypsin digestion and longer half-life (63 h) compared to the native enzyme (33 h). However, none of these new bioconjugates have undergone clinical trials yet.

In 2017, an Irish biopharmaceutical company has entered into a license agreement on the PASylation® Technology to develop a longer-acting ASNase. The PASylation is based on polypeptides composed of Pro, Ala and, alternatively, Ser (PAS), which, under physiological conditions, lack an ordered structure and form a random coil with surprisingly similar biophysical properties as PEG. Furthermore, due to the chemically inert methyl (Ala) and trimethylene (Pro) side groups, these polypeptides lack any side chain reactivity (Ahmadpour and Hosseinimehr, 2018; Gebauer and Skerra, 2018).

**Figure 4:**
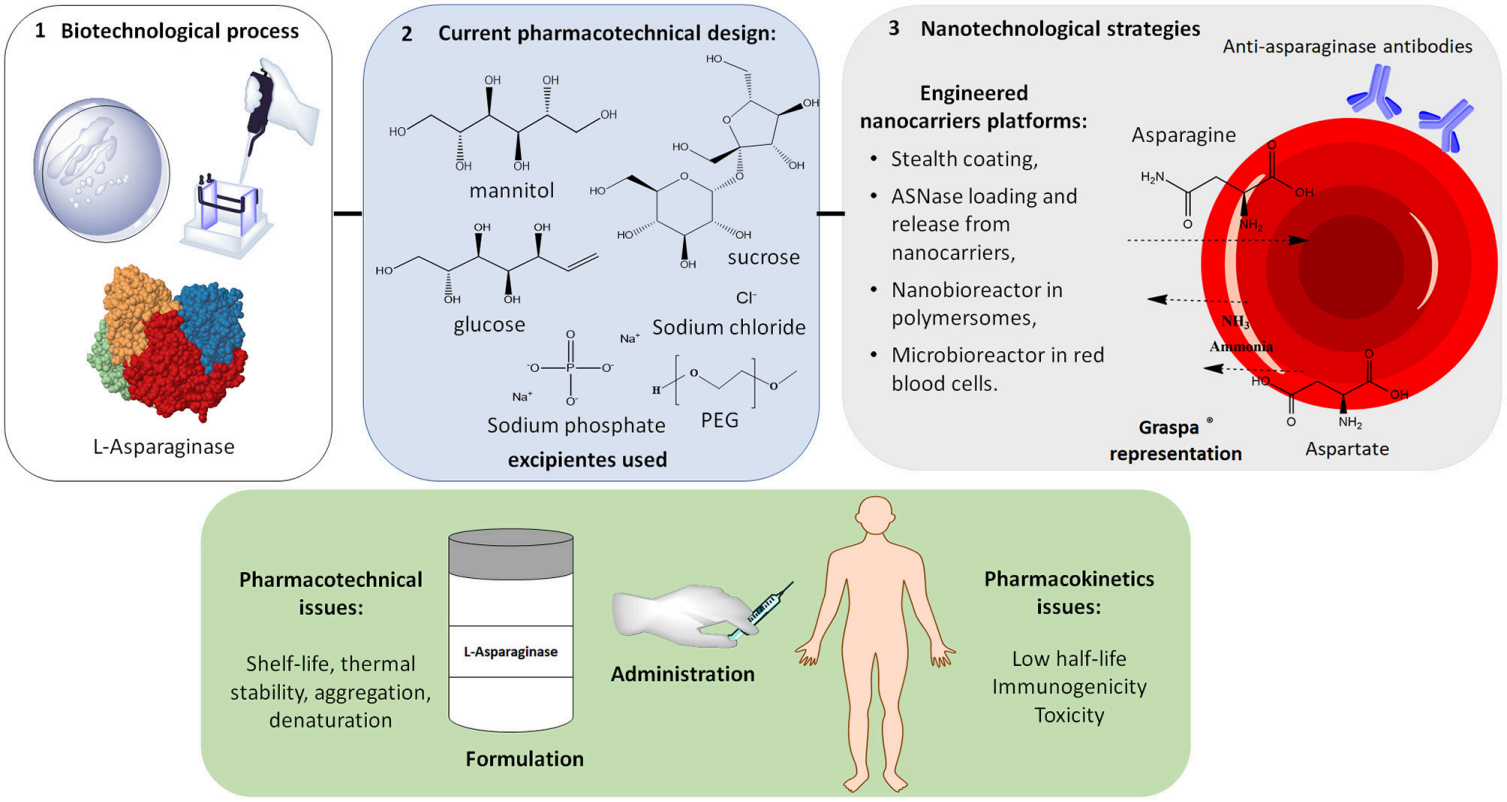
Pharmaceutical issues about L-Asparaginase development as a biopharmaceutical.

Two novel PEGylated ASNases are being developed: PEG-crisantaspase (Asparec®) and Calaspargase Pegol, as second-generation PEGylated drugs. In 2018, phase II and III clinical trials of PEG-crisantaspase were started and, as reported previously, their aim is to administer this biopharmaceutical as a second line therapy in cases of hypersensitivity to the *E. coli* ASNase. Recently, the FDA accepted the Biologics License Application (BLA) for a novel biobetter ASNase: Calaspargase Pegol. In this case, the SS linker of the reactive PEG employed for ASNase PEGylation was replaced by a succinimidyl carbamate linker, creating a more stable bioconjugate. In Figure 4, some of the pharmaceutical issues of ASNase are summarized, while the recombinant and PEGylated ASNase currently undergoing clinical trials are summarized in Table 4.

**Table 4:** Current clinical trials of new recombinant and pegylated L-asparaginase.

**Phase**	**Study**	**Status**		**Country**
**RECOMBINANT L-ASPARAGINASE**				
Phase II	PK, PD, Safety and Immunogenicity of Spectrila in Adults With Acute B-cell Lymphoblastic Leukemia	Not yet recruiting	Estimated Study Completion on July 31, 2021	Brazil
	Trial of Oncaspar® and Three Doses of Pegylated Recombinant Asparaginase in Adult Patients With Newly Diagnosed Acute Lymphoblastic Leukemia	Terminated	Actual Study Completion Date on May 2013	Germany
	Efficacy and Safety of Recombinant Asparaginase in Infants (<1 Year) With Previously Untreated Acute Lymphoblastic Leukemia	Completed	Actual Study Completion Date on February 2011	Germany
Phase III	Comparative Efficacy and Safety of Two Asparaginase Preparations in Children With Previously Untreated Acute Lymphoblastic Leukemia	Completed	Actual Study Completion Date on October 2012	Netherlands
**PEGYLATED L-ASPARAGINASE**				
Phase II	Randomized Study of Intravenous Calaspargase Pegol (SC-PEG Asparaginase) and Intravenous Oncaspar in Children and Adolescents With Acute Lymphoblastic Leukemia or Lymphoblastic Lymphoma	Active, not recruiting	Last Update Posted on September 27, 2017	United States
	A Dose Escalation Phase I Study of Asparec® (mPEG-R-Crisantaspase) Administered as Intravenous (IV) Infusion in Patients With Relapsed or Refractory Hematological Malignancies	Completed	Estimated Study Completion on February 2015	France

*Not yet recruiting: The study has not started recruiting participants*.

*Recruiting: The study is currently recruiting participants*.

*Enrolling by invitation: The study is selecting its participants from a population, or group of people, decided on by the researchers in advance. These studies are not open to everyone who meets the eligibility criteria but only to people in that particular population, who are specifically invited to participate*.

*Active, not recruiting: The study is ongoing, and participants are receiving an intervention or being examined, but potential participants are not currently being recruited or enrolled*.

*Suspended: The study has stopped early but may start again*.

*Terminated: The study has stopped early and will not start again. Participants are no longer being examined or treated*.

*Completed: The study has ended normally, and participants are no longer being examined or treated (that is, the last participant's last visit has occurred)*.

*Withdrawn: The study stopped early, before enrolling its first participant*.

*Unknown: A study on ClinicalTrials.gov whose last known status was recruiting; not yet recruiting; or active, not recruiting but that has passed its completion date, and the status has not been last verified within the past 2 years*.

## Concluding Remarks

Since its initial discovery as a drug in the early 1950' by J. G. Kidd and collaborators, ASNase has become one of the cornerstones of chemotherapeutic treatments, especially of acute lymphoblastic leukemia (ALL) (Kidd, 1953). Recent studies also point toward its potential for the treatment of solid tumors (Knott et al., 2018). Despite of the groundbreaking medical innovation of the first ASNase therapy and its success in extending the lives of millions of people over the last decades, most of the products currently in the market lack desirable pharmaceutical characteristics. Those include, but are not limited to, an extended blood serum half-life as well as reduced immunogenicity and toxicity.

The development of improved ASNases is not an easy task, given the microbiological source of the enzyme, which can result in immunogenicity. In addition to that, serum proteases may degrade the enzyme causing further loss of activity and increased immunogenicity due to additional exposition of epitopes after cleavage. To face those challenges, researchers have nowadays a wide array of biomolecular and biochemical tools at their disposal to aid in the improvement of ASNase, tailoring the enzyme for its application.

The development of biobetter ASNases starts at the process development. This is in line with the classical QbD maxim “*to begin process development with the end* (the product) *in mind*” (Juran, 1992). Following this idea, several research groups have looked into new microbiological sources of ASNases, with special focus on the eukaryotic ones such as *Saccharomyces cerevisiae, Aspergillus sp*. and *Proteus vulgaris*, which might deliver ASNases with reduced immunogenic potential. In accordance to the same guiding principle, genetically engineered and/or chemically modified ASNases have also been investigated, with special attention to site-directed mutation, PEGylation and nanoencapsulation. As J. M. Juran so wisely put it: “*the product is the process*” and some of the product's immunogenicity results from the presence of contaminating biomolecules and protein aggregates, both of which should be removed in the purification steps. Therefore, novel purification strategies are also worth investigating.

Thus, as demonstrated in this review, drawing inspiration from QbD principles, new technologies have been applied to improve the ASNase quality profile. However, there is a need for a deeper understanding of the mechanisms involved in the treatment with ASNase in order to create new and effective strategies to improve this biopharmaceutical.

## Author Contributions

All of the authors contributed significantly to the final version of the present text, since there is a strong integration of the research group. That said, the work was initially conceived by LB, FdS under supervision of AJ, which also composed sections (Introduction) and (Concluding remarks). LB was responsible for most of the literature research presented in the sections (L-Asparaginase) and (Upstream). Section (Biobetters and Biosimilars) was mostly structured and written by LB with contributions from JS. Section (Quality-by-Design) was written by FdS with significant aid from BB. Section (Downstream) was mostly researched and written by EK with later additions from LB and JS. Section (Protein engineering for improvement of L-asparaginase therapeutic use) was conceived and researched by TC-S with later corrections and contributions from GM. Sections (Immobilization), (Nonaencapsulation) and (Current trends in the development of novel biobetter L-asparaginases) was researched and written by AA with significant corrections from CR-Y. Section (PEGylation) was composed by JS and improved upon by CR-Y. The final correction and English proofing were undertaken by AJ, BB, CR-Y, GM, LB, and FdS.

### Conflict of Interest Statement

The authors declare that the research was conducted in the absence of any commercial or financial relationships that could be construed as a potential conflict of interest.
